# Geometrical Characterisation of a 2D Laser System and Calibration of a Cross-Grid Encoder by Means of a Self-Calibration Methodology

**DOI:** 10.3390/s17091992

**Published:** 2017-08-31

**Authors:** Marta Torralba, Lucía C. Díaz-Pérez, Margarita Valenzuela, José A. Albajez, José A. Yagüe-Fabra

**Affiliations:** 1 Centro Universitario de la Defensa, Ctra. Huesca s/n, Zaragoza 50090, Spain; martatg@unizar.es; 2 I3A, University of Zaragoza, C/María de Luna 3, Zaragoza 50018, Spain; jalbajez@unizar.es (J.A.A.); jyague@unizar.es (J.A.Y.-F.); 3 Departamento de Ingeniería Industrial, Universidad de Sonora, Rosales y Blvd. Luis Encinas s/n, Hermosillo 83000, Mexico; margarita.valenzuela@unison.mx

**Keywords:** nanopositioning, 2D-stage, self-calibration, calibration, plane mirror laser interferometer, grid encoder, uncertainty

## Abstract

This article presents a self-calibration procedure and the experimental results for the geometrical characterisation of a 2D laser system operating along a large working range (50 mm × 50 mm) with submicrometre uncertainty. Its purpose is to correct the geometric errors of the 2D laser system setup generated when positioning the two laser heads and the plane mirrors used as reflectors. The non-calibrated artefact used in this procedure is a commercial grid encoder that is also a measuring instrument. Therefore, the self-calibration procedure also allows the determination of the geometrical errors of the grid encoder, including its squareness error. The precision of the proposed algorithm is tested using virtual data. Actual measurements are subsequently registered, and the algorithm is applied. Once the laser system is characterised, the error of the grid encoder is calculated along the working range, resulting in an expanded submicrometre calibration uncertainty (k = 2) for the X and Y axes. The results of the grid encoder calibration are comparable to the errors provided by the calibration certificate for its main central axes. It is, therefore, possible to confirm the suitability of the self-calibration methodology proposed in this article.

## 1. Introduction

The nanotechnology field has grown over recent decades, and the importance of nanotechnology has rapidly increased with the demand for more accurate positioning systems and larger working ranges [1,2]. Positioning systems that operate at a nanometre scale are fundamental in devices used in nanotechnology applications such as nanomanufacturing machine tools or measuring machines. The performance and usage of these machines depend directly on the accuracy of these positioning systems and their working ranges [3]. Within this line of research, a novel 2D nanopositioning platform (NanoPla) has been developed [4,5] with a large working range (50 mm × 50 mm) and a submicrometre uncertainty.

Several sensor technologies currently exist that are suitable for addressing nanopositioning issues. Long-range positioning stages can integrate either 1D or 2D encoders or laser interferometer systems to provide displacement feedback. Spurious motions in out-of-plane positions are typically measured by short-range devices such as capacitive or inductive sensors [2,6]. As for the NanoPla, a laser sensor scheme based on plane mirror laser interferometers has been selected as the most suitable positioning sensor system. In large working ranges, these sensor systems achieve excellent accuracy and ensure direct traceability. When working at a nanometre scale, the accuracy of the positioning sensor system is crucial. The International Organisation for Standardisation (ISO) defines accuracy as the description of random and systematic errors [7]. Random errors can be quantified by taking numerous measurements and processing the resulting data by calculating averages and standard deviations in order to determine the measurement uncertainty. However, systematic errors can be corrected by using calibration methods; this is one of the goals of this paper.

When addressing error compensation techniques, several methods can calibrate positioning sensor systems depending on the type of sensors used, the accuracy required, the size of the working range and the available equipment. Direct calibration methods essentially measure a calibrated artefact in order to calculate the difference between the known and the measured position as the systematic error [8]. However, this procedure has a significant disadvantage when working at a submicrometre scale due to the difficulty in finding a reliable calibrated artefact. This artefact should be more accurate than the system to be calibrated, which can be complicated and costly when working at such a small scale. Self-calibration methods provide an alternative option [9] by relating views of a non-calibrated artefact; a calibrated artefact is not required, thus providing a clear advantage [10]. The only parameter that cannot be obtained through self-calibration is the absolute 1D length scale, which must be set independently.

This article focuses on the geometrical characterisation of a two-dimensional laser system used for X-Y positioning with submicrometre uncertainty. Even though the laser heads have already been separately calibrated by the manufacturer, the correction of the geometric errors existing between the laser detector heads and plane mirrors arrangement is required, especially for this 2D setup. The procedure proposed is based on self-calibration methods, and a non-calibrated artefact is therefore required. This study uses a commercial grid encoder as a non-calibrated artefact. A grid encoder is a 2D positioning sensor that has multiple applications in metrology and precision engineering; it utilises a scanning head that reads a geometric pattern (grid) encoded in a plate. The resulting accuracy is a function of the reference pattern quality. The grid encoder is partially calibrated; the manufacturer provides a calibration certificate with information on the measurement error along the main X and Y axes. When considering the various applications of the grid encoder, knowing its error not only along the main axes but also along the entire 2D working range is beneficial. By applying the proposed self-calibration procedure, the geometric errors of the 2D laser system setup are obtained, and, therefore, its measurements are corrected. Next, by comparing them to the grid encoder measurements obtained from the self-calibration procedure at the same positions, the measurement and squareness errors of the grid encoder can be calculated along the working range. The uncertainty of the laser system measurements can thus be determined after the grid encoder calibration is performed, making it possible to determine the calibration uncertainty of the grid encoder. A similar issue was addressed by Kim et al. in [11], where a self-calibration method was also applied to calibrate a 2D positioning system. However, the approach in this study is different because Kim et al. used additional capacitive sensors to perform the self-calibration.

This work initially describes the methods and materials used. The mathematical model of the 2D laser system is then analysed. This identifies the factors that must be calculated by means of the self-calibration algorithm to correct the laser system geometrical errors. The algorithm is initially tested using virtual data, thus verifying its performance within the working range. Once the actual measurements of the laser system and the grid encoder have been obtained experimentally, the geometric errors of the laser system arrangement are calculated. After the laser system has been geometrically characterised and the readouts corrected, the grid encoder error is calculated at each point evaluated. The calibration uncertainty of the grid encoder is then analysed. Finally, the results of the 2D laser system self-calibration and the error map of the grid encoder are illustrated in order to demonstrate the suitability of the self-calibration proposed methodology.

## 2. Description of the Methods and Materials Used for the Procedure

This work is based on a previous study [12] in which a 2D laser scheme was used as a reference system to characterise 2D grid encoder performance at different temperatures. The metrology frame used was manufactured in a very low thermal expansion glass ceramic material (Zerodur). The laser system was mathematically aligned by using the orthogonality of the grid encoder as the reference. Therefore, the squareness errors of the grid encoder were neglected. In contrast, the present study utilises a self-calibration of the 2D laser sensor system by using the grid encoder as an auxiliary element, and any squareness errors are explicitly considered in the algorithm. To verify this new procedure, a calibration is performed using an experimental setup where the laser system and the non-calibrated artefact are mounted on the same metrology frame as in [12]. The 2D laser system belongs to the Renishaw RLE10 laser interferometer family. It consists of a laser unit (RLU), two sensor heads (RLD), two plane mirrors (one per axis), and an environmental control unit (RCU). In addition, an external interpolator is used to reduce the expected resolution of the system from 10 nm to 1.58 nm. The non-calibrated artefact is a KGM 181 cross-grid encoder (Heidenhain GmbH) with a circular working range of 140 mm in diameter. The calibration is performed along an area equal to the working range of the NanoPla (50 mm × 50 mm).

**Figure 1:**
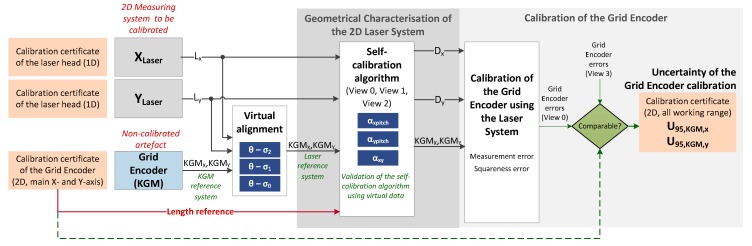
Scheme of the work presented in this article.

Figure 1 represents a scheme of the work presented through the different sections of this article. The laser system is encompassed by two laser heads, which have been previously calibrated by the manufacturer. These laser heads are placed perpendicularly to each other in the setup that is described in the next part of this Section. To correct the geometrical errors of this 2D assembly (see Section 3), a self-calibration procedure was performed. A grid encoder is the non-calibrated artefact used, which has been calibrated by the manufacturer exclusively along its main axes. The laser system and the grid encoder take measurements simultaneously along a mesh of points in the considered working range, for different views of the grid encoder. The grid encoder readouts are mathematically aligned to the laser reference system, and then the self-calibration algorithm described in Section 4 is applied. Once the results are obtained (see Section 5), the geometric errors of the 2D laser system are compensated, and the corrected laser system readouts are used to calibrate the grid encoder along the whole working range. Additionally, the calibration uncertainty of the grid encoder is also calculated (see Section 6). Finally, the errors of the grid encoder along the main axes obtained after this calibration are compared to the ones provided by its calibration certificate.

In Figure 1, $L_{x}$ and $L_{y}$ represent the laser readouts in X and Y axes, respectively. Similarly, KGM_x_ and KGM_y_ represent the KGM 181 cross-grid encoder readouts, and $D_{x}$ and $D_{y}$ are the real displacements. View 0, View 1 and View 2 are the different views used for the self-calibration and View 3 is an additional view measured only for the verification, and that was not used for the self-calibration procedure. ${\theta -\sigma }_{j}$ is the angular deviation between laser system and grid encoder in View j, for j = 0, 1, 2. Finally, $\alpha_{\mathrm{xpitch}}$, $\alpha_{\mathrm{ypitch}}$ and $\alpha_{\mathrm{xy}}$ are the geometric error of the 2D laser system. These parameters will be explained in detail in the following sections of this article.

**Figure 2:**
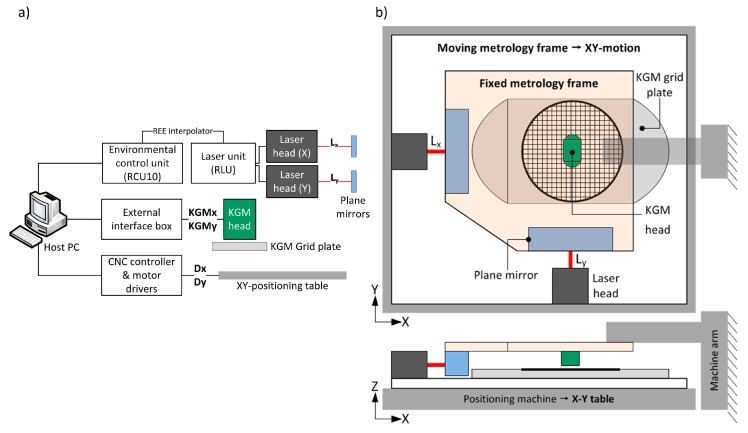
(**a**) Block diagram of the experimental setup connections; (**b**) Sketch of the experimental setup for the calibration procedure.

In a calibration performed at a submicrometre resolution, the design of the experimental setup is crucial, because it can significantly affect the accuracy of the final result. Figure 2a is a block diagram of the connections between devices in the setup and Figure 2b represents a sketch of the setup used for the procedure. As it is shown in Figure 2a, the laser system and the grid encoder are connected to a host PC that records their readouts at each position. An auxiliary positioning machine is used to perform the X-Y displacements.

**Figure 3:**
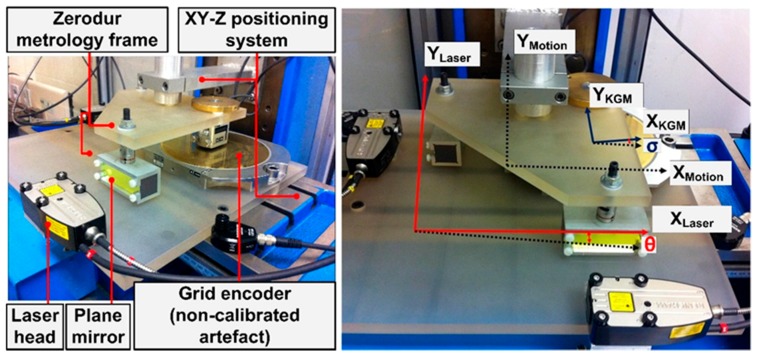
Experimental setup for the calibration procedure: main components (**left**); and defined geometric relationships between reference systems of the 2D sensors (**right**).

The metrology frame consists of two thermally stable parts with different relative motion. The plane mirrors and the grid encoder scanning head are installed in the upper metrology frame, which is attached to the arm of the positioning machine and remains static during the tests. The laser detector heads and the grid base of the 2D encoder are fixed to the lower metrology frame, which is placed on the X-Y table of the positioning machine. To minimise Abbe errors in the X and Y axes, the grid encoder scanning head and the X and Y laser sensors are aligned both in Z and, in advance, at the central position of the measuring range at the XY plane. Figure 3 shows photographs of the experimental setup.

Figure 3 (right) shows the following geometric relationships between the laser system, the grid encoder and the positioning machine for this arrangement: the angular deviation between the reference axes of the positioning machine and the laser system (θ), and the angular deviation between the reference system of the positioning machine and the encoder (σ). The angular deviation between the grid encoder and the laser system is the difference between θ and σ. The values of these angles depend on the assembly and are not expected to be higher than ±0.01 rad.

## 3. Mathematical Model of the 2D Laser System

**Figure 4:**
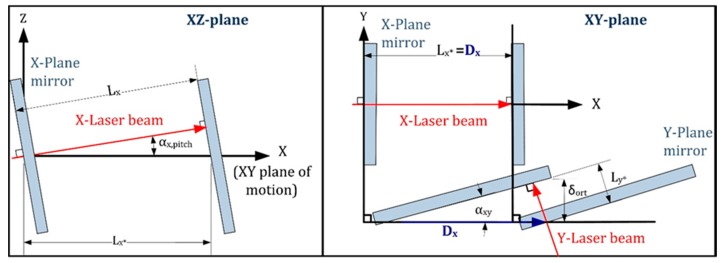
Geometric model of the 2D laser system.

This section analyses the mathematical model of the 2D laser system. The laser system essentially consists of two beams and two plane mirror interferometers (one per axis) that are theoretically orthogonal to each other and coplanar with the X-Y plane of movement. Based on this laser arrangement, two different geometric errors must be compensated. The first is a 1D error present in every plane mirror interferometer; it appears when the laser beam is not coplanar to the X-Y plane of motion. This study refers to this as pitch error (i.e., $\alpha_{\mathrm{xpitch}}$, $\alpha_{\mathrm{ypitch}}$ shown in Figure 4). The second is a 2D error caused by the non-orthogonality of the X and Y plane mirror interferometers ($\alpha_{\mathrm{xy}}$ in Figure 4). This study refers to this as squareness error, similar to [11]. As shown in Figure 4, the model assumes that the incident and reflected laser beams are coincident lines. In other words, each laser beam can be considered orthogonal to its respective plane mirror. This simplifies the problem by making it possible to describe it in a 2D level. This assumption is justified because the manufacturer defines a tight alignment tolerance between the laser beam and the normal vector of the plane mirror (±25 arc s). It applies to both pitch and yaw between laser beam and plane mirror. Considering 100 mm as the maximum possible distance between the mirror and the laser head in the setup, any deviations caused by the orthogonality error between the laser beam and plane are negligible (<<1 nm).

In Figure 4, the angle $\alpha_{\mathrm{xpitch}}$ represents the pitch error in the X-Z plane; similarly, there is a $\alpha_{\mathrm{ypitch}}$ contained in the Y-Z plane. The angle $\alpha_{\mathrm{xy}}$ represents the non-orthogonality between the X and Y plane mirrors, i.e., the squareness error. $D_{x}$ and $D_{y}$ are the actual displacements of the moving part, whereas $L_{x}$ and $L_{y}$ are the laser readouts of the X and Y axes, respectively. In Figure 4, there is a real displacement of the plane mirrors in the X direction ($D_{x}\neq 0$), but in the Y direction there is no movement ($D_{y}=0$). However, after correcting the pitch error of the Y laser interferometer readout ($L_{y*}$), the squareness error ($\delta_{\mathrm{ort}}$) persists and requires correction. The geometric relations of the model are listed below, where the measurement errors in the X and Y axes are represented by $\delta_{x}$ and $\delta_{y}$, respectively.

(1) $$D_{x}{=L}_{x*}=\frac{L_{x}}{\cos\left(\alpha_{\mathrm{xpitch}}\;\right)}$$

(2) $$D_{y}=\frac{L_{y*}}{{\cos(\alpha }_{\mathrm{xy}})}{+\delta }_{\mathrm{ort}}=\frac{L_{y}}{\cos\left(\alpha_{\mathrm{ypitch}}\;\right){\cos(\alpha }_{\mathrm{xy}})\;}{+\delta }_{\mathrm{ort}}$$

(3) $$\delta_{\mathrm{ort}}=\frac{{\tan(\alpha }_{\mathrm{xy}}{)L}_{x}}{\cos\left(\alpha_{\mathrm{xpitch}}\;\right){\cos(\alpha }_{\mathrm{ypitch}})\;}$$

(4) $$\delta_{x}{=D}_{x}-L_{x}=\left(\frac{1}{\cos\left(\alpha_{\mathrm{xpitch}}\;\right)}-1\right)L_{x}$$

(5) $$\delta_{y}{=D}_{y}-L_{y}=\left(\frac{1}{\cos\left(\alpha_{\mathrm{ypitch}}\;\right){\cos(\alpha }_{\mathrm{xy}})\;}-1\right)L_{y}{+\delta }_{\mathrm{ort}}$$

Therefore, the target values that should be obtained through the self-calibration are $\alpha_{\mathrm{xpitch}}$, $\alpha_{\mathrm{ypitch}}$ and $\alpha_{\mathrm{xy}}$. Once these angles are known, it is possible to correct the laser system readouts.

## 4. Self-Calibration Procedure

Performing a calibration at a submicrometre scale presents difficulties. The most significant difficulty is determining a calibrated artefact at least one order of magnitude more accurate than the target accuracy of the laser system—ideally, the same size as the working range. The application of self-calibration techniques can solve this problem [9], thus making self-calibration a suitable procedure for optimising the performance of nanopositioning systems in a large working area [13,14]. A method based on reversal techniques is selected as the best option for this study, where the goal is to obtain submicrometre uncertainty along the working range.

A self-calibration procedure essentially consists of a non-calibrated artefact with measurement features whose geometrical relationships remain invariable. These features are measured by the system and calibrated by using different views. Because the non-calibrated artefact is assumed to be a rigid body, the pattern of the measurement features remains invariant over the different views [15]. The only calibrated pattern required is a 1D-length scale to correct the scale factor of the system to be calibrated [9]. To address this inconvenience, other authors have proposed a calibrated reference rod [16]. In the case of this study, the wavelength of the laser beam has already been calibrated by the manufacturer. Therefore, it is not necessary to correct the scale factor of the laser system. Nevertheless, a 1D scale reference is needed to measure the displacements of the grid encoder in the translation views, as it will be explained in the following subsection.

At any point, the observed error is, then, an addition of the system error and the non-calibrated artefact error. By combining the different equations for the three views, it is possible to isolate the laser system error. As a novelty, this paper selects a different approach based on the use of a measuring instrument as a non-calibrated artefact. In the following, the self-calibration algorithm is explained in detail, and its performance is verified using virtual data.

### 4.1. Definition of the Self-Calibration Algorithm

The case in this study is very specific, as the non-calibrated artefact is a grid encoder that uses a measuring grid and a scanning head instead of measuring features to provide X-Y coordinates. The input data, then, become the readouts of the laser system and the grid encoder at the same positions. Nevertheless, these measurements are taken in different reference axes. Hence, these two axes must be virtually aligned to allow a comparison of both measurements. A measurement from point to point along each of the main axes of the grid encoder can be corrected by using the information from its calibration certificate. This measurement can be used during the calibration as an absolute length.

In a self-calibration procedure, the global error at each position is expressed as the addition of the positioning error of the calibrated system plus the intrinsic errors of the non-calibrated artefact plus the alignment errors of the non-calibrated artefact at each view. In this case, the alignment errors are not present because the grid encoder readouts are previously mathematically aligned to the laser system axes. For the initial view (View 0), the global error $V_{0}$ at each point is expressed as in Equation (6):

(6) $$V_{0}{(x}_{i}{,\;y}_{j})=M_{0}{(x}_{i}{,\;y}_{j})+E_{0}{(x}_{i}{,\;y}_{j})$$

where $M_{0}$ is the laser system positioning error at the point ${(x}_{i},\;y_{j})$ at its own reference axes, and $E_{0}$ is the grid encoder positioning error at the point ${(x}_{i},\;y_{j})$ rotated to the laser system reference axes (θ-σ, in Figure 3) and displaced to the laser system origin. The grid has N × N points, thus i, j = 1, 2, …, N.

As it can be observed in Equation (6), once the laser system positioning errors, $M_{0}$, are calculated by the self-calibration procedure, the grid encoder positioning errors, $E_{0}$, can also be deduced (Section 6).

**Figure 5:**
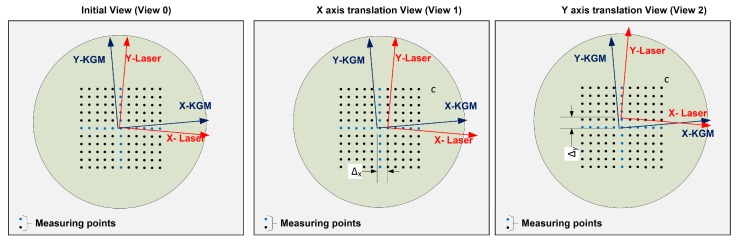
Schematic of the initial and translation views, including the reference systems and their geometric relationships and the mesh of the measuring points.

According to the mathematical model presented in Section 3, three factors must be calculated by the self-calibration algorithm. At least three different views of the artefact are required to cancel the systematic errors of the non-calibrated artefact. In this particular case, a system of three equations is obtained by combining the expressions of the initial view and two additional translation views. Therefore, three different positions of the grid encoder are sufficient to perform this calibration (see Figure 5): the initial view (View 0), the X axis translation view (View 1) and the Y axis translation view (View 2). View 1 is obtained by moving the grid encoder approximately 5 mm ${(\Delta }_{x})$ along its X axis, and View 2 is achieved by moving the grid encoder approximately 5 mm ${(\Delta }_{y})$ along its Y axis (see Figure 5). The real value of these displacements ($\Delta_{x}$ and $\Delta_{y}$) needs to be known, for this purpose, the calibrated main axes of the grid encoder are used. Given this particular setup, the relative position between the laser system and the positioning machine remains invariant for all the views as does the angular deviation θ between the positioning machine and the laser system. The relative position between the grid encoder and the positioning machine changes when the encoder is moved. Hence, the angular deviation σ between the positioning machine and the grid encoder may be slightly different for View 0, View 1 and View 2. As previously noted, the non-calibrated artefact provides coordinates for each measuring point. The displacement to reach the positions of each measuring point is given by the positioning machine that has been programmed to measure a mesh of points covering the entire working range. Due to the errors of the positioning machine and the variation of the σ angle from View 0 to View 1 and View 2, the points measured in each view may not be exactly the same. However, it has been observed that the maximum deviation between the points in different views is 0.05 mm. According to the calibration certificate of the grid encoder, nearby points have similar error values (with differences in the range of a few nanometres). Thus, it is possible to assume that the artefact errors can be compensated by relating the views.

A mesh of 11 × 11 points separated 5 mm from each other is measured for each view covering the working range. By combining Equations (4)–(6), the Expressions (7)–(10) are obtained, where $V_{0x}$ and $V_{0y}$ are the global errors for View 0 in X and Y axes, respectively. Similarly, $V_{1x}$, $V_{1y}$ and $V_{2x}$, $V_{2y}$ are the global errors in X and Y axes, for View 1 and View 2, respectively. In every case, the global errors of each view ($V_{0},V_{1},V_{2}$) are experimentally obtained. $\delta_{x}$ and $\delta_{y}$ are the laser system errors in X and Y axes. Likewise, $E_{x}$ and $E_{y}$ are the non-calibrated artefact errors in X and Y axes.

(7) $$V_{0x}{(x}_{i}{,\;y}_{j}{)=\delta }_{x}{(x}_{i}{)+E}_{x}{(x}_{i}{,\;y}_{j})$$

(8) $$V_{0y}{(x}_{i}{,\;y}_{j}{)=\delta }_{y}{(x}_{i}{,\;y}_{j}{)+E}_{y}{(x}_{i}{,\;y}_{j})$$

(9) $$V_{1x}{(x}_{i}{+\Delta }_{x}{,\;y}_{j}{)=\delta }_{x}{(x}_{i}{+\Delta }_{x}{)+E}_{x}{(x}_{i}{,\;y}_{j})$$

(10) $$V_{1y}{\;(x}_{i}{+\Delta }_{x}{,y}_{j}{)=\delta }_{y}{(x}_{i}{+\Delta }_{x}{,y}_{j}{)+E}_{y}{(x}_{i}{,\;y}_{j})$$

(11) $$V_{2x}{(x}_{i}{,\;y}_{j}{+\Delta }_{y}{)=\delta }_{x}{(x}_{i}{)+E}_{x}{(x}_{i}{,\;y}_{j})$$

(12) $$V_{2y}{(x}_{i}{,y}_{j}{+\Delta }_{y}{)=\delta }_{y}{(x}_{i}{,y}_{j}{+\Delta }_{y}{)+E}_{y}{(x}_{i}{,\;y}_{j})$$

View 0 and View 1 provide two linearly independent equations each, and View 2 just one because Equations (7) and (11) are linearly dependent, that is 5 linearly independent equations in total: (7)–(10) and (12). There are 5 unknown variables: the geometric errors $\alpha_{\mathrm{xpitch}}$, $\alpha_{\mathrm{ypitch}}$ and $\alpha_{\mathrm{xy}}$ and the systematic encoder errors $E_{x}$ and $E_{y}$. Therefore, three views are enough to perform the calibration. It is worth noting that the squareness error of the laser system $\alpha_{\mathrm{xy}}$ is decoupled from the squareness errors of the grid encoder that is considered in its systematic error E(x, y).

The global error at the initial view, consisting of the positioning error of the laser system and the grid encoder at a particular point, is compared to the global error at the translation view, consisting of the positioning error of the encoder at the same point and the positioning error of the laser system at a point displaced a distance equal to Δ. Therefore, the positioning errors of the grid encoder are cancelled, but not the ones of the laser system. The positioning errors of the laser system, $\delta_{x}$ and $\delta_{y}$, are replaced by Equations (1)–(3). Finally, considering the following property, Equations (13)–(15):

(13) $$L_{x}{(x}_{i}{+\Delta }_{X})-L_{x}{(x}_{i}{)=L}_{x}{(\Delta }_{x})$$

(14) $$L_{y}{(x}_{i}{+\Delta }_{x}{,\;y}_{j})-L_{y}{\;(x}_{i}{,\;y}_{j}{)=L}_{y}{(\Delta }_{x},\;0)$$

(15) $$L_{y}{(x}_{i}{,\;y}_{j}{+\Delta }_{y})-L_{y}{\;(x}_{i}{,\;y}_{j}{)=L}_{y}{(0,\;\Delta }_{y})$$

The following equation system of three unknown variables ($\alpha_{\mathrm{xpitch}}$, $\alpha_{\mathrm{ypitch}}$ and $\alpha_{\mathrm{xy}}$) and three equations is obtained:

(16) $$V_{1x}{(x}_{i}^{1}{+\Delta }_{x}{,y}_{j}^{1})-V_{0x}{(x}_{i}^{0}{,y}_{j}^{0})=\left(\frac{1}{\cos\left(\alpha_{\mathrm{xpitch}}\;\right)}-1\right)L_{x}{(\Delta }_{x},\;0)$$

(17) $$V_{1y}{(x}_{i}^{1}{+\Delta }_{x}{,y}_{j}^{1})-V_{0y}{(x}_{i}^{0}{,y}_{j}^{0})=\frac{{\tan(\alpha }_{\mathrm{xy}})}{\cos\left(\alpha_{\mathrm{xpitch}}\;\right){\cos(\alpha }_{\mathrm{ypitch}})\;}L_{x}{(\Delta }_{x},\;0)$$

(18) $$V_{2y}{(x}_{i}^{2}{,y}_{j}^{2}{+\Delta }_{y})-V_{0y}{(x}_{i}^{0}{,y}_{j}^{0})=\left(\frac{1}{\cos\left(\alpha_{\mathrm{ypitch}}\;\right){\cos(\alpha }_{\mathrm{xy}})}-1\right)L_{y}{(0,\;\Delta }_{y})$$

where ${(x}_{i}^{0}{,y}_{j}^{0})$, ${(x}_{i}^{1}{+\Delta }_{x}{,y}_{j}^{1})$ and ${(x}_{i}^{2},y_{j}^{2}+\Delta_{y})$ for i, j = 1, …, 11 are the coordinates of the points measured along the working range in View 0, View 1 and View 2, respectively, while $L_{x}{(x}_{i}{,\;y}_{j})$ and $L_{y}{(x}_{i}{,\;y}_{j})$ are the laser readouts in X and Y axes at those points.

### 4.2. Validation of the Self-Calibration Algorithm

The proposed method should work within all the expected ranges for the angles. Therefore, the values should be calculated with the required precision in order to obtain the actual displacements ($D_{x}$ and $D_{y}$) with submicrometre uncertainty (see Table 1). As it was previously explained, before starting the self-calibration, the algorithm mathematically aligns the grid encoder readouts to the laser system reference axes. As long as the squareness error of the laser system is sufficiently small, this mathematical alignment does not have a significant effect on the estimation of the geometrical errors of the laser system. By mathematical simulations, the acceptable range has been defined to be ±1 × 10^−2^ rad. In order to achieve submicrometre uncertainty in the 2D positioning, $\alpha_{\mathrm{xy}}$ should be determined with an error of ±1 × 10^−6^ rad. However, for the setup used for the experiment, the orthogonal error of the laser system, $\alpha_{\mathrm{xy}}$ was measured and corrected using a CMM. The maximum accuracy achieved during the setup depends directly on this CMM and it was ±5 × 10^−3^ rad. Therefore, the proposed self-calibration procedure is needed to determine the value of $\alpha_{\mathrm{xy}}$ with an uncertainty of ±1 × 10^−6^ rad. With regards to pitch errors, the proposed self-calibration procedure is able to calculate and correct them, even if the range of $\alpha_{\mathrm{xpitch}}$ and $\alpha_{\mathrm{ypitch}}$ is unbounded, independently of their value. To obtain the desired uncertainty, they should be determined with an error of ±1 × 10^−3^ rad.

Before beginning the experiment and measuring actual data, the proposed algorithm is validated using virtual data. To create the virtual data, the readouts of the grid encoder and the laser system at the same positions are simulated along the working range of 50 mm × 50 mm. The actual positions ($D_{x}$ and $D_{y}$) are initially defined in a mesh of 11 × 11 points. The positioning machine movement is simulated with steps of 5 mm from −25 mm to +25 mm along the X and Y axes, covering the entire working range. The grid encoder readouts include linear errors of [5 × 10^−5^]·$D_{x}$ and [5 × 10^−5^]·$D_{y}$ in the X and Y components, respectively. The laser system readouts ($L_{x}$ and $L_{y}$) are obtained according to the geometric relations of Figure 4 by selecting values inside the expected range for $\alpha_{\mathrm{xpitch}}$, $\alpha_{\mathrm{ypitch}}$ and $\alpha_{\mathrm{xy}}$. Readouts for the three views (View 0, View 1 and View 2) are simulated, taking into account that the angle between the reference system of the laser system and the reference system of the grid encoder (θ-σ), which is different for each view because, as previously noted, σ is not constant. In a previous work [4], the random error of the laser system was calculated by considering the laser resolution, the wavelength instability, beam mixing, and environmental influences. The combination of these errors resulted in 7 nm. In this virtual validation of the algorithm, the laser system readouts, as well as the grid encoder readouts, include a random uniform error of 20 nm. The method used to validate the self-calibration algorithm proceeds as follows: firstly, the laser system readouts are corrected using the values obtained for $\alpha_{\mathrm{xpitch}}$, $\alpha_{\mathrm{ypitch}}$ and $\alpha_{\mathrm{xy}}$; secondly, the grid encoder error is isolated and compared to the one set at the beginning; and finally, if both errors are equal the self-calibration algorithm is validated. This simulation verifies that the error of the grid encoder is linear along the X and Y axes as initially set. In addition, by defining different values for the random error, this simulation also provides a clear insight of the good theoretical repeatability of the system, in the range of ±25 nm. Therefore, the algorithm is considered to be valid.

By performing different simulations and including random errors of the expected order in the readouts of the laser system and the grid encoder, it is possible to estimate the expanded uncertainty (k = 2) of the resulting values given by the algorithm. It is observed that the accuracy depends on the value of $\alpha_{\mathrm{xy}}$; when its value is inside the expected acceptable range, the goal of submicrometre uncertainty is achieved (see Table 1).

## 5. Geometrical Characterisation of the Laser System Setup

Self-calibration can be performed once the setup used for the calibration is defined and the self-calibration algorithm has been explained and verified. Before starting the experiments, the stability of the laser system was studied by performing static repeatability tests inside the working range. A total of 9000 readouts were recorded at each point during 60 s. The resulting stability (2σ) was 24 nm, which was considered acceptable. During the calibration procedure, in order to minimise random errors of both, the laser system and the grid encoder, 100 readouts were recorded and averaged at each measured point.

To perform the self-calibration, it is necessary to know the real displacement of the grid encoder in the translation views (View 1 and View 2). The grid encoder has been calibrated by the manufacturer along its central X and Y axes. Therefore, this calibration is used as an absolute length reference to measure these translations in X and Y axes. The readouts of the laser system and the grid encoder are subsequently taken at the same time and the same positions in the defined mesh of 11 × 11 points. Once all the points are measured, the data obtained are the coordinates of 121 points in two different reference systems (the laser system and the grid encoder) and three different views (View 0, View 1 and View 2). Initially, the self-calibration algorithm mathematically aligns both reference systems; by applying Equations (16)–(18), the algorithm cancels the grid encoder systematic error and obtains the correction factors ($\alpha_{\mathrm{xpitch}}$, $\alpha_{\mathrm{ypitch}}$ and $\alpha_{\mathrm{xy}}$) as well as the angle between the reference system of the laser system and the grid encoder (θ-σ) for each View. The obtained results can be seen in Table 2. They are given with the required uncertainty specified in Section 2 and $\alpha_{\mathrm{xy}}$ is inside the expected range of ±5 × 10^−3^ rad. To obtain the actual displacements ($D_{x}$ and $D_{y}$), it is necessary to correct the laser system readouts ($L_{x}$ and $L_{y}$) by applying the correction factors ($\alpha_{\mathrm{xpitch}}$, $\alpha_{\mathrm{ypitch}}$ and $\alpha_{\mathrm{xy}}$) into Equations (1) and (2).

## 6. Calibration of the Grid Encoder

As previously explained, the grid encoder calibration certificate compares the central axes with the measurement of a calibrated laser interferometer. These calibrated axes have been used as a length reference to calculate the real displacements of the grid encoder in X and Y axes, for View 1 and View 2, before starting the self-calibration procedure. However, the grid encoder is used in many applications that require a high accuracy not only in the central axes but throughout the entire grid. Some approaches for grid calibrations can be found in the literature, as the one presented in [17]. This work proposes an alternative solution to this issue; once the readouts of the laser system are corrected, it is possible to compare them with the grid encoder readouts in order to calculate the error of the grid encoder at each measured point. Thereby, the systematic error of the non-calibrated points that were not part of the main axes will be known. Additionally, this procedure validates the effectiveness of the self-calibration by comparing the error obtained for the central X and Y axes with the error provided in the calibration certificate by the manufacturer.

It is important to note that, even if the systematic errors of the grid encoder were present in Equations (7) to (12), they were cancelled by reversal techniques. Thus, they were not present in Equations (16) to (18), which were applied in the self-calibration algorithm. Once the laser system readouts are corrected, it is possible to use them to calculate the error of the grid encoder readouts.

The following sections initially explain how the grid encoder is calibrated and confirm the effectiveness of the self-calibration of the laser system. The error map of the grid encoder is then presented, and the uncertainty of its calibration calculated.

### 6.1. Calibration Procedure of the Grid Encoder Using the Laser System

**Figure 6:**
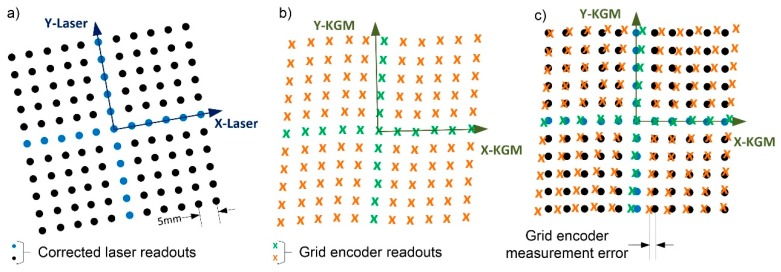
(**a**) Mesh of points measured by the laser system after correction; (**b**) Mesh of points measured by the grid encoder; (**c**) Their alignment and comparison.

Initially, the proposed procedure mathematically aligns the corrected laser readouts with the reference system of the grid encoder; the grid encoder error is then calculated as the difference between the two measurements. The rotation angle to align both reference systems is θ-σ (see Figure 3), as calculated in the previous section by means of an optimisation process. The grid encoder measurements may be affected by the grid squareness errors in addition to linear errors; therefore, the rotation angle is calculated by matching the X axis of the encoder to the X axis of the laser system, taking the orthogonality error of the grid encoder to its Y axis. For this reason, the Y component of the grid encoder errors is higher than the X component. This situation is illustrated in Figure 6. The mesh of points measured by the laser system after correction (Figure 6a) exhibits neither squareness nor linear errors, but the mesh of points measured by the grid encoder exhibits both (Figure 6b). Once the two meshes are aligned, and because the laser system measurements have already been corrected through self-calibration, the difference between the coordinates of the same point measured by the grid encoder and the laser system is the grid encoder error (see Figure 6c). By comparing these two meshes, it is also possible to determine the squareness error of the grid encoder.

**Figure 7:**
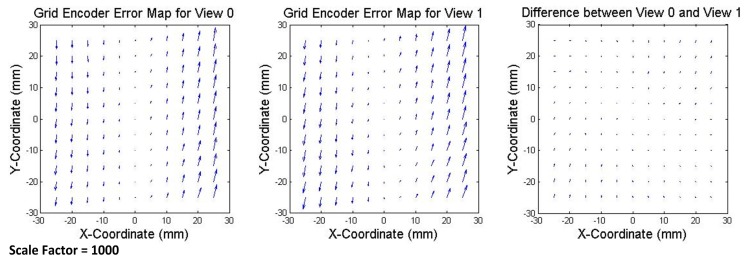
Error map of the grid encoder in View 0 (**left**); View 1 (**center**); and their comparison representing the difference (**right**). Scale factor equal to 1000.

The error of the grid encoder can be calculated by using the readouts of the initial view (View 0) or the translation views (View 1 and View 2). The results should be very similar in all the cases. However, it is expected that they are not coincident since the random errors of the laser system and the random error of the grid encoder are still present. In addition, as previously stated, the measuring points are slightly different in each view. The error maps of the grid encoder calculated for View 0 and View 1 and the difference between the two error maps are represented in Figure 7. It can be observed that both maps have similar error trends and orders of magnitude (the three maps use the same scale factor, equal to 1000), and in both cases, the error is smaller near the central axes. These presented results are similar to the error map calculated for View 2.

**Figure 8:**
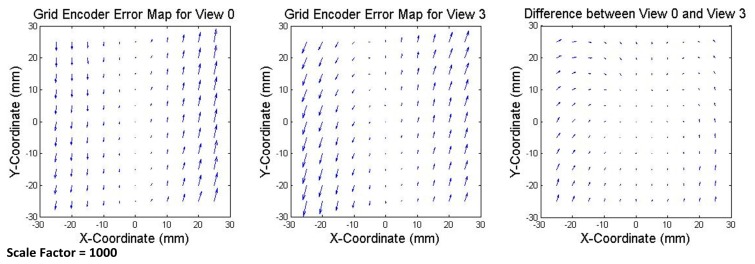
Error map of the grid encoder in View 0 (**left**); View 3 (**center**); and their comparison representing the difference (**right**). Scale factor equal to 1000.

To validate the calibration procedure, the errors of the grid encoder are also calculated in an additional view, named View 3, which was not used during the self-calibration procedure. View 3 is only used for this verification. The grid encoder errors should be similar to the ones calculated for the previous views because the grid encoder errors are independent of the views. In View 3, the grid encoder was rotated 180 degrees around its Z axis, and similarly to the other views, the measurements were taken simultaneously by the grid encoder and the laser system along the same mesh of 11 × 11 points separated 5 mm from each other. Because the setup of the laser system remains invariant, the readouts can be corrected using the parameters calculated in the self-calibration. In Figure 8, the error maps of the grid encoder in View 0 and View 3 are represented and compared (the three maps use the same scale factor, equal to 1000). It can be observed, as in the previous case, that both error maps have similar trends and orders of magnitude and the difference is due to the random errors of the laser system and the grid encoder and to the fact that the measured points are not the same in both views.

The squareness error of the grid encoder calculated for View 0 is almost coincident with that calculated for View 1, View 2 and View 3: 1.36 × 10^−4^ rad. The resulting squareness error is very small and can be considered negligible in this working range with a submicrometre uncertainty.

**Figure 9:**
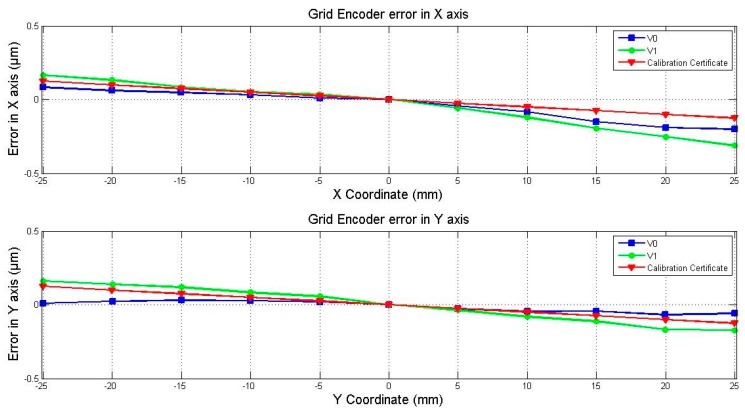
Error of the grid encoder along the main X and Y axes in the analysed range.

The calibration certificate information includes the error along the two main axes in a range of 140 mm. The middle point, where the error is equal to zero, is coincident with the zero position in the experimental self-calibration, where the error is also equal to zero. The X component of the grid encoder error along the central X axis and the Y component of the error along the Y axis for View 0 and View 1 are represented in Figure 9. They can be easily compared to each other and to the error given by the calibration certificate of the grid encoder, also represented in Figure 9. The error provided by the calibration certificate is represented only for the working range of the laser system (±25 mm). It can be seen that the errors calculated experimentally for View 0 and View 1 and the errors provided by the calibration certificate are highly comparable, with the same linear trend and order of magnitude. Therefore, the self-calibration procedure for the grid encoder calibration can be considered validated.

### 6.2. Calibration Uncertainty of the Grid Encoder

The error of the grid encoder at each point is calculated as the average between the resulting errors of View 0, View 1 and View 2. The expanded uncertainty of the calibration system (U_95_) is evaluated according to [18]. All the error sources and their contributions to the uncertainty of the system are examined, and the uncertainty is calculated with a coverage factor of k = 2, thus providing a level of confidence of approximately 95%, as shown in Equation (19).

(19) $$U_{\mathrm{KGM}}(k=2)=k\sqrt{u_{\mathrm{laser}}^{2}{+u}_{T}^{2}+\frac{S_{\mathrm{Cal}.\mathrm{KGM}}^{2}}{n_{\mathrm{Cal}.\mathrm{KGM}}}{+u}_{\mathrm{KGMResolution}}^{2}{+u}_{\mathrm{ResidualError}}^{2}}$$

The standard uncertainty is calculated as the square root of the quadratic sum of the contributions of the uncertainty sources. These sources are listed below:

- The uncertainty of the calibration system, i.e., the laser sensors, represented as $u_{\mathrm{laser}}$.
- The uncertainty of the expansion and contraction of the grid encoder due to small changes in the temperature $u_{T}$.
- The repeatability of the grid encoder measurements during the calibration $S_{\mathrm{Cal}.\mathrm{KGM}}$. The term $n_{\mathrm{Cal}.\mathrm{KGM}}$ represents the number of repetitions of each measurement.
- The uncertainty of the grid encoder resolution $u_{\mathrm{KGMResolution}}$.
- The error that could not be corrected by the calibration is $u_{\mathrm{ResidualError}}$ and is dependent on the distance of the calibrated point to the main central axes.

The uncertainty of the laser system is calculated according to Equation (20); its contributors are listed below:

(20) $$U^{2}{}_{\mathrm{Laser}}{=u}_{\mathrm{estimatedLaser}}^{2}+\frac{S_{\mathrm{Laser}}^{2}}{n_{\mathrm{Laser}}}{+u}_{\mathrm{mirrors}}^{2}{+u}_{\mathrm{LaserResolution}}^{2}{+u}_{\mathrm{SelfCalibration}}^{2}$$

- The standard uncertainty of the laser according to its manufacturer, denoted as the estimated uncertainty $u_{\mathrm{estimatedLaser}}$.
- The repeatability of the laser measurements $S_{\mathrm{Laser}}$. The term $n_{\mathrm{Laser}}$ represents the number of repetitions of each measurement.
- The uncertainty of the flatness of the mirrors $u_{\mathrm{mirrors}}$.
- The uncertainty of the resolution of the laser $u_{\mathrm{LaserResolution}}$.
- The uncertainty of the self-calibration procedure of the laser system $u_{\mathrm{SelfCalibration}}$.

The estimated uncertainty of the laser system, as well as the uncertainty of the flatness of the mirrors and the laser resolution, were previously calculated in [12] for the same laser system. Therefore, the same values can be applied to this calculation. The repeatability of the laser system depends on the measurements taken during the calibration procedure of the grid encoder and is thus particular to this study. The uncertainty of the self-calibration procedure depends on the uncertainty of the calculation of the correction factors. As previously mentioned, a submicrometre uncertainty is achieved after the calibration; the uncertainty of the laser system measurements after applying the self-calibration is ±25 nm.

Once the uncertainty of the laser system is calculated, it is possible to proceed with the calculation of the expanded calibration uncertainty of the grid encoder. The calculation of the laser system uncertainty is represented in Table 3, and the contributions of the uncertainty sources of the grid encoder calibration and the resulting value are represented in Table 4.

As shown in Table 3, the expanded uncertainty (k = 2) is $U_{95,x}[\mathrm{nm}]=\;226+4\;x\;[\mathrm{mm}]$ and $U_{95,y}[\mathrm{nm}]=\;410+15\;y\;[\mathrm{mm}]$ for errors in the X and Y axes, respectively, in a working range of ±25 mm. The variables x and y represent the distance of the calibrated point to the X or Y main axes, respectively, and must be introduced in millimetres. The calibration certificate specifies that the position error along the entire range of the grid encoder (a circular area 140 mm in diameter) is ±2 μm, so it can be assumed that the position error inside the area of study (±25 mm) is approximately 0.8 μm. It is thus confirmed that the calibration procedure of the grid encoder performed in this study reduced the uncertainty of the grid encoder measurement. This reduction was achieved because the calibration was performed at every point of the mesh and the uncertainty sources were examined in detail.

## 7. Discussion and Conclusions

This study defined a self-calibration method and applied to the geometrical characterisation of a two-dimensional laser sensor system. Self-calibration eliminates the difficulty of finding a calibrated artefact with better accuracy than the system to be calibrated because the calibration is performed using a non-calibrated artefact. In this study, the non-calibrated artefact was a grid encoder. Thus, once the laser system was geometrically characterised, it was also possible to use it to calibrate the grid encoder.

The setup and the mathematical model of the 2D laser system were analysed in order to establish the geometrical errors that needed to be calculated through the self-calibration. The self-calibration algorithm and the setup were described in detail. After applying the calibration algorithm and correcting the readouts of the laser system, it was possible to determine the measurement error of the grid encoder at each measured point of the calibrated range by comparing the corrected laser readouts and the grid encoder readouts, obtained from the previous application of the self-calibration procedure. The calibration uncertainty of the grid encoder calibration was calculated, thus obtaining a result of $U_{95,x}[\mathrm{nm}]=\;226+4\;x\;[\mathrm{mm}]$ and $U_{95,y}[\mathrm{nm}]=410+15\;y[\mathrm{mm}]$ for errors in the X and Y axes, respectively. The suitability of the self-calibration procedure was verified in different ways in this work. First, the proper performance of the procedure was checked by using virtual data. Second, the experiment was carried out, and actual data were measured. The measurements were taken in three different views. Thus, once the laser system readouts were corrected, the grid encoder error map was calculated by using the readouts of the three views, verifying that the grid encoder errors remain invariant independently of the view. The error map of the grid encoder of one of the views used for the self-calibration procedure was compared to the error map calculated for an additional view that was not part of the self-calibration procedure, in order to validate the experimental procedure. Though the maps were not the same, they exhibited the same trend and order of magnitude. Their small differences could be due to random errors of the laser system and the grid encoder. Finally, in order to confirm the proper function of the self-calibration procedure with a different approach, the error calculated along the central axes was compared to the calibration certificate error; as expected, both were linear errors with the same trend and order of magnitude. Therefore, the self-calibration procedure described in this paper could be considered as valid and presents many advantages over direct calibration.

## Figures and Tables

**Table 1:** Factors of the considered 2D geometric model calculated by the self-calibration algorithm.

Parameter	Required Uncertainty	Description
$\alpha_{\mathrm{xy}}$	±1 × 10^−6^ rad	Squareness error
$\alpha_{\mathrm{xpitch}}$	±1 × 10^−3^ rad	Pitch error in X-axis
$\alpha_{\mathrm{ypitch}}$	±1 × 10^−3^ rad	Pitch error in Y-axis
θ-σ	±1 × 10^−4^ rad	Angular deviation between the reference systems of the grid encoder and the laser system

**Table 2:** Geometric parameter results of the experimental self-calibration procedure and virtual alignment.

Parameter	Required Uncertainty	Resulting Value	Description
$\alpha_{\mathrm{xy}}$	±1 × 10^−6^ rad	4.010 × 10^−3^ rad	Squareness error
$\alpha_{\mathrm{xpitch}}$	±1 × 10^−3^ rad	9 × 10^−3^ rad	Pitch error in X-axis
$\alpha_{\mathrm{ypitch}}$	±1 × 10^−3^ rad	10 × 10^−3^ rad	Pitch error in Y-axis
${\theta -\sigma }_{0}$	±1 × 10^−4^ rad	−4.9 × 10^−3^ rad	Angular deviation between the reference systems of the grid encoder and the laser system
${\theta -\sigma }_{1}$	±1 × 10^−4^ rad	−4.6 × 10^−3^ rad	
${\theta -\sigma }_{2}$	±1 × 10^−4^ rad	−4.6 × 10^−3^ rad	

**Table 3:** Calculation of the standard uncertainty of the laser system.

Uncertainty Source	Justification	X-Axis Contribution [nm]	Y-Axis Contribution [nm]
Estimated uncertainty of the laser system	According to the manufacturer, for a distance of 60 mm	76	76
Laser repeatability	Calculated for the readouts of this experiment	6	3
Planarity of the mirrors	According to the manufacturer	63	63
Laser resolution	Resolution of 1.58 nm, achieved by external interpolators	1.58/√12	1.58/√12
Self-calibration procedure	Self-calibration algorithm maximum error = ±25 nm, according to the virtual validation	25/√3	25/√3
**Standard uncertainty of the laser system**		**99**	**99**

**Table 4:** Calculation of the expanded calibration uncertainty (U95) of the grid encoder.

Uncertainty Source	Justification	X-Axis Contribution [nm]	Y-Axis Contribution [nm]
Laser system	Calculated in Table 3	99	99
Thermic variation	Thermal variation of ±0.40 °C during the experiment	48	48
Grid encoder repeatability	Calculated for the readouts of this experiment	5	3
Grid encoder resolution	Resolution of 1 nm	1/√12	1/√12
Residual error [nm]	Calculated by comparing the grid encoder error obtained in View 0, 1 and 2	24.5 + 2 x[mm] * * It is dependent on the position in X-axis expressed in mm	173 + 7.5 y[mm] ** ** It is dependent on the position in Y-axis expressed in mm
**U95 of the grid encoder [nm]**		226 + 4 x[mm]	410 + 15 y[mm]
